# Landscape Analysis of Adult Florida Panther Habitat

**DOI:** 10.1371/journal.pone.0133044

**Published:** 2015-07-29

**Authors:** Robert A. Frakes, Robert C. Belden, Barry E. Wood, Frederick E. James

**Affiliations:** 1 U.S. Fish and Wildlife Service, South Florida Ecological Services Office, 1339 20th Street, Vero Beach, Florida, United States of America; 2 National Park Service, South Florida Natural Resources Center, Everglades National Park, Homestead, Florida, United States of America; Fenner School of Environment and Society, AUSTRALIA

## Abstract

Historically occurring throughout the southeastern United States, the Florida panther is now restricted to less than 5% of its historic range in one breeding population located in southern Florida. Using radio-telemetry data from 87 prime-aged (≥3 years old) adult panthers (35 males and 52 females) during the period 2004 through 2013 (28,720 radio-locations), we analyzed the characteristics of the occupied area and used those attributes in a random forest model to develop a predictive distribution map for resident breeding panthers in southern Florida. Using 10-fold cross validation, the model was 87.5 % accurate in predicting presence or absence of panthers in the 16,678 km^2^ study area. Analysis of variable importance indicated that the amount of forests and forest edge, hydrology, and human population density were the most important factors determining presence or absence of panthers. Sensitivity analysis showed that the presence of human populations, roads, and agriculture (other than pasture) had strong negative effects on the probability of panther presence. Forest cover and forest edge had strong positive effects. The median model-predicted probability of presence for panther home ranges was 0.81 (0.82 for females and 0.74 for males). The model identified 5579 km^2^ of suitable breeding habitat remaining in southern Florida; 1399 km^2^ (25%) of this habitat is in non-protected private ownership. Because there is less panther habitat remaining than previously thought, we recommend that all remaining breeding habitat in south Florida should be maintained, and the current panther range should be expanded into south-central Florida. This model should be useful for evaluating the impacts of future development projects, in prioritizing areas for panther conservation, and in evaluating the potential impacts of sea-level rise and changes in hydrology.

## Introduction

The Florida panther (*Puma concolor coryi*) is a subspecies of puma (also called mountain lion or cougar). Pumas were once widely distributed throughout North and South America, but have been extirpated from the eastern United States except for a small breeding population of Florida panthers in southern Florida (in this paper, we use the term “puma” when referring to the species as a whole and “panther” when referring specifically to the Florida panther subspecies). Panthers, like all pumas, are wide ranging, secretive, and occur at low densities. They require large contiguous areas to meet their social, reproductive, and energetic needs [1], a requirement that is being compromised by rapid development in southern Florida. Panther habitat continues to be lost to urbanization, residential development, conversion to agriculture, and mining [1]. Highways result in loss and fragmentation of habitat, lead to traffic-related panther mortality, and encourage further human development [2]. Urban, suburban, and exurban areas eliminate, fragment, and alter panther habitat and increase the potential for panther-human interactions. The recovery strategy for the Florida panther includes: (1) maintaining, restoring, and expanding the panther population and its habitat in southern Florida; (2) expanding this population into south-central Florida if sufficient habitat exists; (3) establishing at least two additional viable populations within the historic range outside of south and south-central Florida; and (4) facilitating panther recovery through public awareness and education [1]. The keystone to this recovery strategy is the existing panther population in southern Florida. Because habitat loss, degradation, and fragmentation are among the greatest threats to this population, there is a need for land use planning that incorporates panther conservation and recovery.

Several resource selection analyses have been completed to identify habitats selected by panthers in southern Florida. Following the ordering of selection processes suggested by Johnson [3], the majority of these analyses were third order selections (habitat within home ranges) [4–9], 3 were second order selection analyses (home ranges within the range) [6, 7, 10], and only Kautz et al. [7] and Thatcher et al. [10] attempted to analyze the first order selection (range within the region). Kautz et al. [7] identified areas that had been consistently occupied by panthers for 20 years (“Primary Zone”), adjacent areas that would be most likely to be occupied by an expanding panther population (“Secondary Zone”), and areas that would best facilitate dispersal and population expansion north of the Caloosahatchee River (“Dispersal Zone”). Thatcher et al. [10] developed a panther habitat model using the Mahalanobis distance statistic and landscape characteristics within panther home ranges, based on older (mid-1990s) telemetry and landscape data. Since these studies were completed, a great deal of new land use/land cover information and panther telemetry data have become available.

Recovery Action 1.1.4.2. in the Third Revision of the Florida Panther Recovery Plan [1] calls for updating the Kautz et al. [7] map every five years. The objective of this study was to develop a first-order predictive, landscape-scale model based on occurrence data to predict the distribution of Florida panther habitat to meet this requirement. Our study differs from most previous work in that it was intended to examine the large-scale mixture of landscape characteristics where panthers are found, as opposed to distances of panther locations from specific habitat patches, as used in most previous studies. The model will be useful in evaluating the impacts of future development projects, prioritizing areas for panther conservation (e.g., mitigation areas, panther conservation banks, conservation easements, and fee title purchases), identifying areas outside the study area for possible panther reintroductions, and evaluating the potential impacts of sea-level rise and changes in hydrology.

## Methods

### Study area

**Fig 1:**
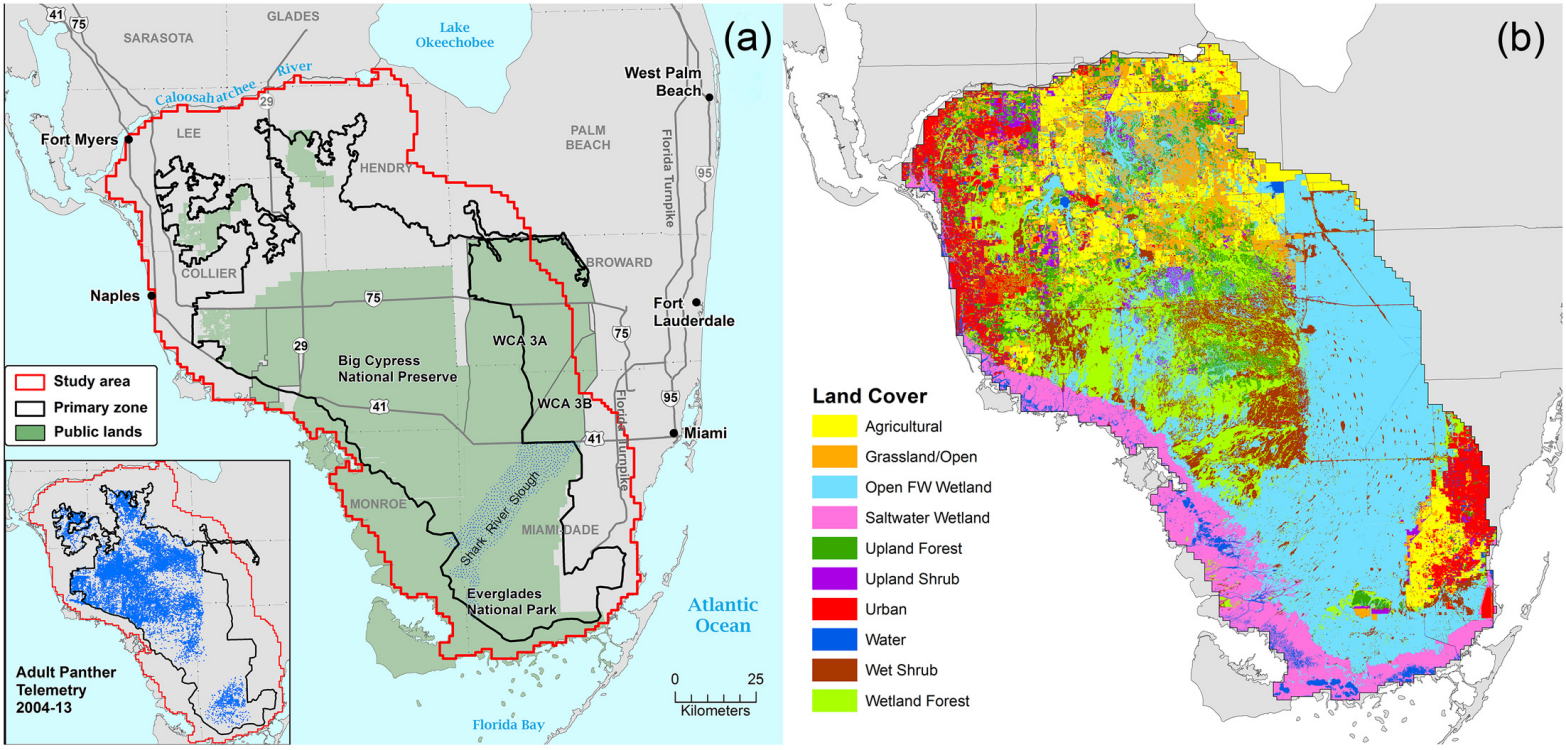
Location of the Florida panther study area and major land cover classes. (a) Main map shows the study area in relation to the Primary Zone, an area of focus by conservation agencies. Inset shows the location of resident adult telemetry points from 2004 through 2013. Breeding panthers do not occur north of the Caloosahatchee River. (b) Geographical distribution of 10 major land cover categories within the study area, used as explanatory variables in the random forest model. Categories were distilled from the Florida Land Use and Cover Classification System (FLUCCS).

The study area (Fig 1a) was located in southwest Florida where the only breeding population of Florida panthers occurs. The study area was designated by drawing an approximate 16-km buffer (roughly the width of an average female home range) around the Primary Zone described by Kautz et al. [7]. This area is bordered on the west and south by the Gulf of Mexico and Florida Bay, on the north by the Caloosahatchee River, and on the east by the 16-km buffer drawn around the Primary Zone boundary. Near-shore islands within the 16-km buffer were excluded. The 16,678 km^2^ study area included most of Everglades National Park, Big Cypress National Preserve, Florida Panther National Wildlife Refuge, Fakahatchee Strand Preserve State Park, and other public lands, as well as thousands of acres of undeveloped land in private ownership. It also contained large agricultural and urbanized areas, the latter including Naples, Fort Myers, and the outskirts of Miami. The study area was divided into 16,678 square kilometer grid cells (1.0 km on each side). This grid size was chosen to account for telemetry error (within 124–230 m [4, 8, 11]) and because of our interest in analyzing panther habitat characteristics at the landscape scale.

### Panther telemetry data

Methods for collecting telemetry locations have been described elsewhere [4, 8, 11]. Our radio-telemetry dataset consisted of all locations collected from February 1981 through June 2014 (*n* = 103,828) as part of ongoing research and monitoring by the Florida Fish and Wildlife Conservation Commission and the National Park Service [12]. During this 33-year monitoring program, 228 panthers were radio-collared and each collared individual was relocated 3 times per week, if possible, during the entire time that it wore a functioning collar. During the time frame of this study (2004–2013), an estimated average of 44% (range 26–62%) of the known population of resident adult panthers was collared and monitored each year, based on annual panther counts by McBride et al. [13]. Of all radio-locations collected, 102,818 locations were within the study area. These data were filtered to the period from January 2004 through December 2013 to be contemporaneous with the data for cover type, roads, human population, and water depth. To avoid including dependent kittens and young transient males, only data for breeding-age panthers (≥3 years old) [14] were utilized. After applying the above filters, only individuals with ≥50 telemetry points were included, in order to reduce the effects of small sample sizes on home range estimates [15]. Home ranges were plotted as 100% minimum convex polygons using the convex hull tool in ArcMap version 10.2.2 (ESRI, Redlands, CA). The final filtered dataset consisted of 87 adult panther home ranges (52 females and 35 males) comprising 28,720 telemetry locations (18,124 for females and 10,596 for males) (Fig 1a). Because of the large number of animals monitored, the frequency of relocations, and the fact that panther home ranges are large and overlap extensively, we felt that areas used and avoided by adult panthers during the 10-year time frame of this study could be accurately identified using these locations within a small margin of error (see below). Therefore, grid cells containing at least one telemetry point from the filtered dataset were classified as “present;” all others were classified as “absent.”

### Landscape variables

#### Land cover types

Vegetation cover types and land uses were obtained from the Florida Land Use and Cover Classification System (FLUCCS) Geographic Information System (GIS) database [16]. There were 76,609 FLUCCS polygons in the study area representing 124 different vegetation cover/land use classes. We combined these into 10 major land cover categories (Table 1). Categories were selected based on our judgment of characteristics important to panthers, such as amount of cover provided (forest, shrub, open), human disturbance (urban, agriculture), or cover types known to be avoided by panthers (open water, saltwater wetlands). Percentages of each of the 10 major land cover categories in each grid cell were calculated using the area tool in ArcMap, and used as explanatory variables in the model.

**Table 1:** Land cover categories used as explanatory variables and their extent in the study area.

Land cover category	Description	Area (km^2^)	Percent of study area
**Open fresh- water wetland**	Freshwater marsh, sawgrass, and wet prairies	5715.9	34.3
**Wetland forest**	Includes cypress strands and domes, hydric pine flatwoods, cypress-mixed hardwoods, bay swamps, mixed wetland hardwoods, cypress-pine-cabbage palm, and wet melaleuca	2457.2	14.7
**Agriculture**	Croplands including row crops, field crops, sugar cane, citrus groves, ornamentals	1610.1	9.7
**Saltwater wetland**	Mangrove swamps, saltwater marshes, and tidal flats	1474.8	8.8
**Grassland**	Includes improved and unimproved pastures, and herbaceous (dry) prairies	1274.5	7.6
**Wet shrub**	Mixed wetland shrubs	1360.2	8.2
**Urban**	Residential, developed, industrial, commercial, or disturbed lands	1158.1	6.9
**Upland forest**	Includes pine flatwoods, upland hardwood forest (e.g., oak-cabbage palm), and hardwood-coniferous mixed forest	895.1	5.4
**Open water**	Lakes, reservoirs, rivers, bays, canals	379.4	2.3
**Upland shrub**	Shrub and brushland, palmetto prairies, and mixed rangeland	351.2	2.1

#### Other landscape variables

The primary prey species of the Florida panther in southern Florida are white-tailed deer (*Odocoileus virginiana*) and wild hogs (*Sus scrofa*) [17, 18]. The white-tailed deer is an “edge species” and the amount of edge affects both the quality and quantity of deer [19, 20]. Lacking spatial density data for these species, we estimated the amount of forest edge in each grid cell as a possible measure of prey availability (i.e., panther hunting habitat) [21, 22]. Using the 10 cover types described above, we defined forest edge as the line between forest polygons and other cover types considered suitable to form a natural edge. Under this definition, polygons classified as urban or agricultural were not considered to be edge-forming. Grasslands and prairies, bodies of fresh water, shrubs, and open freshwater wetlands were counted as edge-forming where they were adjacent to forest. In addition, deer in Florida preferentially use areas where upland forest is adjacent to swamps [23]. Therefore, the edge between upland forest and wetland forest polygons was also counted as forest edge.

We estimated average wet and dry season water depths for the period 1999–2009 for each grid cell of the study area. The wet season was defined as June through October and the dry season as November through May. The value of the water depth variable represented a long-term average water depth for an entire grid cell. A negative depth implied that most of the water table was below the surface, but did not necessarily indicate a completely dry (upland) cell. Similarly, positive values suggested that most, but not necessarily all, of a cell was wetland.

Creating a water depth surface involved subtracting ground surface elevation from a corresponding stage elevation (water level). Daily mean surface water and groundwater data were acquired from the databases of Everglades National Park [24] and the South Florida Water Management District [25]. Gauging stations both within and exterior to the study area were included to minimize boundary or edge effects when generating seasonal water depths. Ground surface elevations for the southeastern portion of the study area were obtained from the U.S. Geological Survey High Accuracy Elevation Data project (HAED) [26]. Topography data for the northwestern portion of the study area were obtained from the U.S. Army Corps of Engineers [27]. Vertical accuracy ranged from +/- 7.5 to +/- 15.0 cm, depending on the source. Generating the average seasonal water depth required interpolating the stage value between the gauging stations, relating each stage value to a corresponding ground surface elevation, subtracting the ground surface elevation from the corresponding stages, and averaging the multiple water depths in each grid cell to produce a single value for each cell (see S1 Text for details).

We calculated average human population density for each cell in the study area using census block data from the 2010 U.S. Census [28]. Census blocks were intersected with the study area grid to obtain an area-weighted average human density for each cell. We calculated the total length of roads in each cell based on the 2011 TIGER/Line shapefiles of Florida roads [29]. Roads classified as four-wheel drive, bike trails, or pedestrian trails were excluded, because we felt that these did not represent enough disturbance to impact panther use of an area.

### Modeling approach

We used random forest (RF) modeling because of demonstrated advantages of RF over other types of statistical classifiers that include: (1) very high classification accuracy; (2) a method of ranking variables according to their importance; (3) the ability to model complex interactions; and (4) RF makes no assumptions about the distribution of predictor or response variables [30]. We tested many different modeling techniques before selecting RF. These included logistic regression, mixed effects logistic regression, generalized additive models (GAM), negative binomial, and Maxent. We also tested both presence-absence and used-available (resource selection function) designs. Although all of these methods produced similar results, we found RF to be more accurate at predicting known panther locations, to be more straightforward to use, and to provide more useful information than the other methods. Because the emphasis of our modeling effort was on prediction rather than explanation, we felt that predictive accuracy was the most important factor on which to base our selection of a modeling technique.

Our model was based on a presence-absence design, in which grid cells lacking a telemetry location were assumed to be absences. Generally, this assumption is potentially invalid because the species could have occurred at the location during the study but was not detected, thus these locations are often referred to as “pseudo-absences” [31]. However, we considered our panther dataset to be valid (i.e., true absences) and the use of an area by resident adult panthers without being detected highly unlikely. Our reasons for this included the large number of animals monitored (228 total, 87 in this study), long duration of the monitoring program (33 years, 10 years in this study), high frequency of monitoring flights (3 times per week), large size of the cells (1.0 km^2^), and the fact that panther home ranges overlap extensively. The latter is important because, although not all panthers were radio-collared (only about half during the time frame of this study), it is likely that all areas used by panthers contained some collared individuals. In addition, the total area occupied by resident adult panthers in south Florida is relatively small, and the entire area is surveyed each year by expert trackers using hounds (e.g., [13]). Therefore, we believe that a presence-absence study design is appropriate in this case.

The model was run using the randomForest package in R (version 3.1.1, www.r-project.org). The type of random forest was classification, with 500 classification trees generated at each run, and 3 variables tried at each split. The model included 15 explanatory variables: 10 land cover categories (see Table 1), plus forest edge, dry season water depth, wet season water depth, human population density, and road density. Male and female panthers do not select significantly different habitat [8, 9], and our preliminary modeling showed that building separate models for males and females did not improve model accuracy. Therefore, the model was built using combined male and female occurrence data. Model-predicted probabilities of presence (*P*) were used to classify each grid cell as present (i.e., adult panther habitat) or absent (i.e., non-habitat). We classified a grid cell as “present” when the model-predicted probability of presence was ≥ 0.338, because at this cutoff point model sensitivity and specificity were equal. Selecting a cutoff threshold where sensitivity equals specificity tends to approximate the observed prevalence of the species in the study area [31].

The RF model was validated using 10-fold cross validation [32]. The training dataset was randomly divided into 10 equal-sized groupings. Nine of the groups (90%) were then combined and used to construct a model, which was used to classify the remaining group. This process was repeated ten times until all of the groups had been classified. Accuracy metrics from this process were compared with the out-of-bag accuracy of the original model. Accuracy metrics calculated included PCC (percent of cells correctly classified), sensitivity (proportion of “present” cells correctly classified), specificity (proportion of “absent” cells correctly classified), kappa (a measure of improvement of classification accuracy above that expected by chance), and AUC (area under the receiver operating characteristic curve) [31].

Sensitivity analysis consisted of plotting the model’s response to changes in one predictor while holding the other predictors constant, using the Plotmo library (version 2.2.1) in R. Plotmo’s default value for the fixed variables is the median [33]. However, this commonly used approach in which one variable is changed while the others are held at a single value (usually the median or mean) would be inadequate with our model, because some variables had different effects depending on the starting point. For example, increasing some variables might have a negative effect in a landscape that was already good habitat, but a positive effect in poor habitat. Also, assigning all variables a median or mean value was not realistic, because in no case could all variables in a cell be at their central tendency at the same time. In good habitat, beneficial landscape characteristics are high while detrimental ones are low, and vice versa for poor habitat. Therefore, model response to changes in each variable was examined in narrow ranges of *P* values corresponding to excellent panther habitat (*P* = 0.85–0.95), medium habitat (*P* = 0.45–0.55), and poor habitat (*P* = 0.05–0.15). The subset of observed variable values producing *P* values within each range was determined. Averages were calculated for each variable for each range, and these served as the fixed value for sensitivity analysis. This allowed for use of realistic combinations of variable values as opposed to simply using median values.

We calculated the mean probability of presence for each panther home range from the *P* values of the grid cells contained within the home range. Mean probability of presence in home ranges were compared using the Wilcoxon rank sum test.

The randomForest package in R provides two measures of variable importance: (1) mean decrease in model accuracy, determined by randomly permuting one predictor variable at a time and determining the resulting loss in model classification accuracy, and (2) the Gini importance, calculated as the mean decrease in node impurity attributed to each predictor variable [34, 35]. Although both methods are presented here, the former is considered to be the more advanced method [34].

## Results

### Landscape variables

GIS analysis of land cover types showed the study area to be predominantly wetlands (Table 1, Fig 1b). Open freshwater wetlands (mostly sawgrass and freshwater marsh) were by far the largest land cover category, representing more than one third of the study area. Wetland forest and wet shrub lands were also important categories concentrated mainly in the center of the study area. Saltwater wetlands, mainly mangrove swamps, bordered the study area on the east and south. Altogether, wetlands made up 66.0% of the study area. In contrast, natural or semi-natural upland areas (upland forest, shrub lands, and grasslands) comprised only about 15.1% of the total. Upland forest, comprising only 5.4%, was scattered in small patches throughout the northern half of the study area. Urban areas predominated in the extreme northwestern (Naples-Ft. Myers area) and southeastern (Miami area) parts of the study area. Agriculture occurred mainly in the northern one-third of the study area, although there was also a large concentration of crop areas on the eastern border of Everglades National Park.

**Fig 2:**
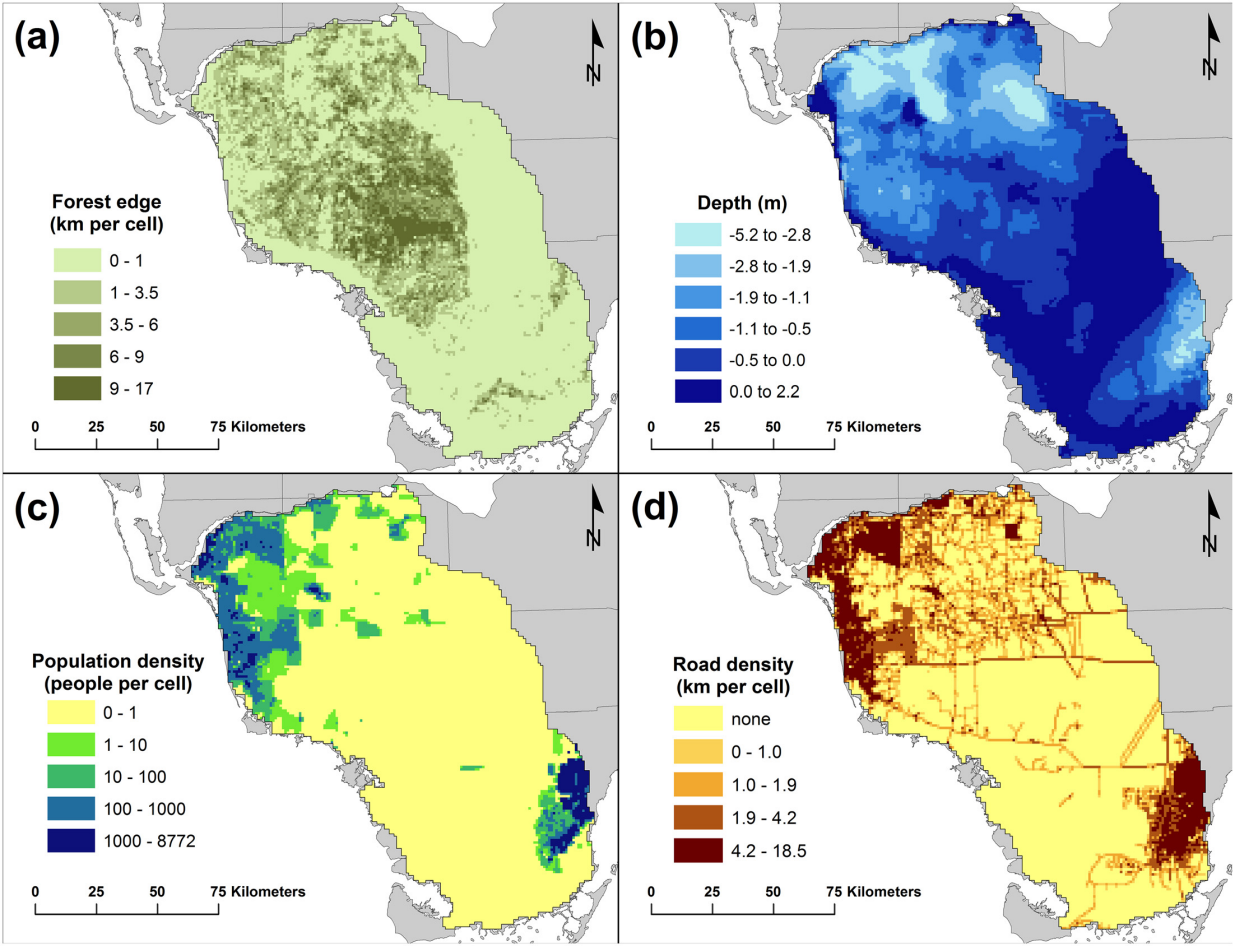
Landscape characteristics within the study area used as explanatory variables. (a) amount of forest edge (km/km^2^); (b) average water depths during the dry season (m); (c) area-weighted average human population density (people/km^2^); (d) road density (km/km^2^).

The amount of forest edge in each cell ranged from essentially none in the vast sawgrass wetlands, coastal areas, and agricultural areas, to 17 km per cell in the center of the study area (Fig 2a). Forest edge was not well-correlated (R^2^ = 0.42) with the total amount of forest cover in a cell, i.e., some areas with low amounts of forest cover might have large amounts of edge, and vice versa.

Average wet and dry season water depths in the study area ranged from -5.2 m (below ground surface) up to +2.6 m above ground. Water depths during the wet season averaged about 0.3 m greater than during the dry season. Wet season and dry season water depths were highly correlated (R^2^ = 0.98). Nevertheless, we chose to keep both variables in the model because removing one of them resulted in a slight loss in model accuracy. The driest areas occurred in the northern and extreme southeastern portions of the study area, corresponding with the well-drained agricultural and residential land uses in those areas. The highest water depths occurred in the Water Conservation Areas, the Shark River Slough in Everglades National Park, and the coastal bays of southwestern Florida. The center of the study area had mostly intermediate water depths (Fig 2b).

Human population density ranged from uninhabited throughout much of the study area, to upwards of 8,000 people per grid cell (km^2^) in the densely populated areas in the northwest and southeast corners of the study area (Fig 2c). Road density showed a similar pattern, ranging from roadless in much of the study area to over 18 km of roads per grid cell in some urban areas. However, even the most undeveloped part of the study area is bisected by several major highways including Interstate 75, US 41, and US 29 and also contains many minor roadways (Figs 1a and 2d).

### Model results

**Fig 3:**
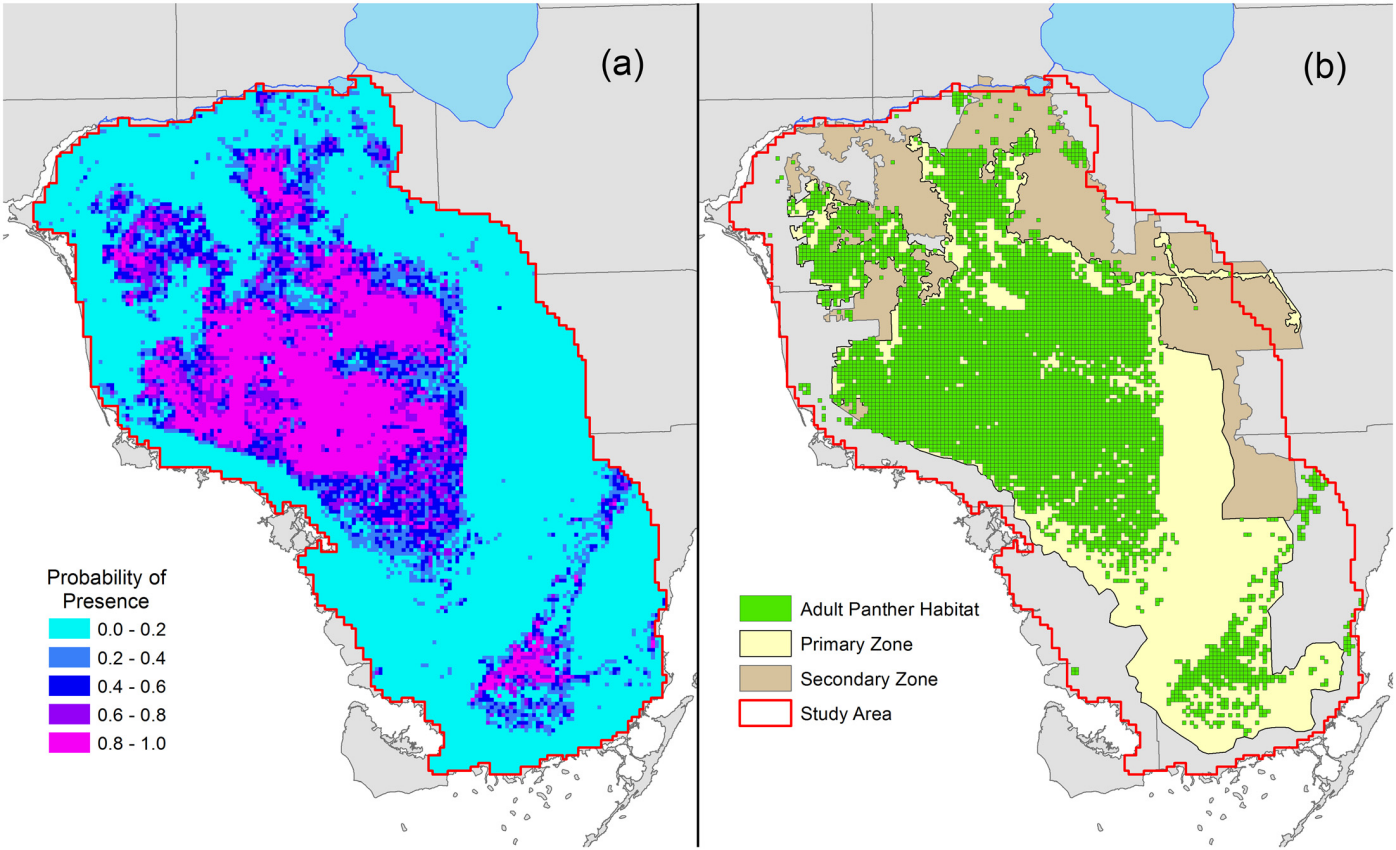
Probability of presence and adult panther habitat. (a) Probability of presence (*P*) of resident adult panthers throughout the study area in south Florida, as predicted by the random forest model. (b) Grid cells with *P* > 0.338 are considered to be adult (breeding) panther habitat. Adult panther habitat is shown in relation to the Primary and Secondary Zones.

Probabilities of panther presence for each grid cell predicted by the model were plotted on a map of the study area (Fig 3a). Adult panther habitat (therefore breeding habitat) was defined as those grid cells classified as “present”, i.e., having a probability of presence of adult resident panthers greater than 0.338 (Fig 3b). Using this cutoff point, 5579 km^2^ of breeding habitat were identified within the study area. Areas of high probability of panther presence were, for the most part, concentrated in a single large contiguous block within the central and northwestern part of the study area. A separate, smaller area of predicted panther use occurred in the southwestern portion of the study area within Everglades National Park. Breeding panther presence was not likely in the Water Conservation Areas, Shark River Slough, or the coastal wetlands of southwest Florida.

#### Accuracy

The RF model accurately predicted the presence or absence of adult panthers in the study area. Using a cutoff probability of 0.338, the RF model had an overall accuracy (PCC) of 87.7% of cells correctly classified, based on out-of-bag error rates (Table 2). By simple resubstitution of the training data, the RF model correctly classified 99% of the grid cells. Sensitivity and specificity were both equal to the PCC at this cutoff point. The kappa statistic (0.711) and AUC (0.95) both indicated high model accuracy in predicting panther presence within the study area. Ten-fold cross validation accuracy was nearly identical to out-of-bag accuracy for all metrics (Table 2).

**Table 2:** Accuracy metrics for the Florida panther habitat model.

Method of Calculation	PCC^a^	Specificity	Sensitivity	Kappa	AUC^b^
**resubstitution**	98.7	98.5	99.1	0.97	1.00
**out-of-bag**	87.7	87.6	87.7	0.71	0.95
**10-fold cross validation**	87.5	87.4	87.7	0.71	0.95

^a^Percent correctly classified.

^b^Area under the receiver operating characteristic curve.

#### Variable importance

**Fig 4:**
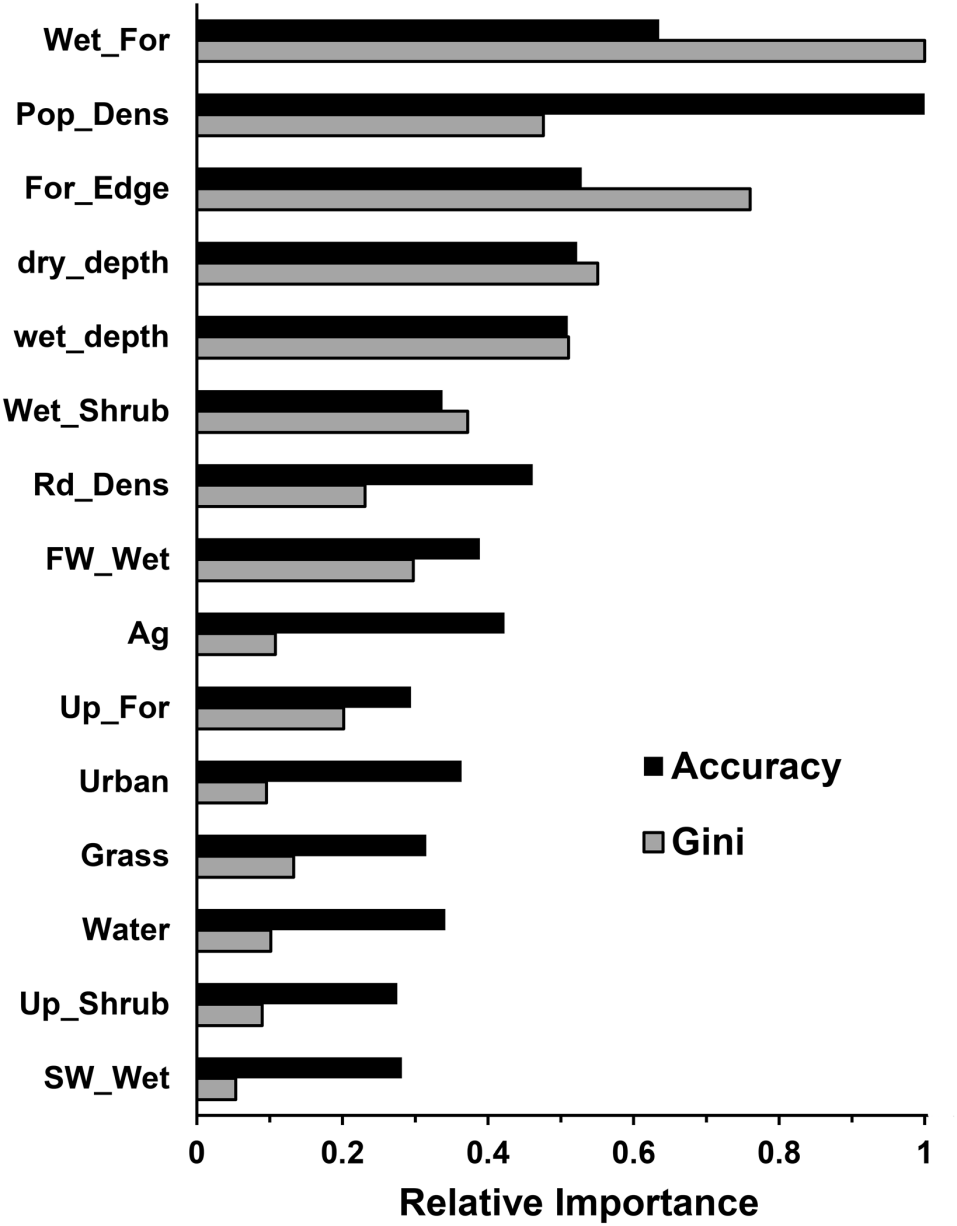
Variable importance. Importance was calculated based on mean decrease in model accuracy (black bars) and mean decrease in Gini index (gray bars). Importance scores were standardized relative to the most important variable by each method. Variables are ranked from highest to lowest importance, based on combined scores from the two methods. Wet_For = wetland forest, Pop_Dens = human population density, For_Edge = forest edge, dry_depth = average dry season water depth, wet_depth = average wet season water depth, Wet_Shrub = wetland shrub, Rd_Dens = road density, FW_Wet = open freshwater wetlands, Ag = agricultural, Up_For = upland forest, Grass = grasslands/dry prairies, Water = open water, Up_Shrub = upland shrub, SW_Wet = saltwater wetland.

The 15 explanatory variables are ranked from highest to lowest importance in Fig 4. Human population density stood out as the most important variable affecting model accuracy, followed by wetland forest. The amount of wetland forest and forest edge were the most important variables according to the Gini index. The top five variables were the same by both importance measures, although in different order. Using the combined relative importance from the two methods, the order of variable importance was wetland forest > human density > forest edge > dry season water depth > wet season water depth. It is surprising that both water depth variables were included in the top five, even though they were highly collinear. Wetland shrubs, road density, freshwater wetlands, and agricultural use were of medium importance relative to the other variables. The upland cover types (upland forests, grasslands, and upland shrubs) did not score as highly in importance as expected. Along with urban, open water, and saltwater wetlands, these were among the least predictive variables. There was greater variation in importance among the variables based on the Gini index compared with model accuracy. According to the accuracy analysis, all variables contributed somewhat to model accuracy.

#### Sensitivity analysis

**Fig 5:**
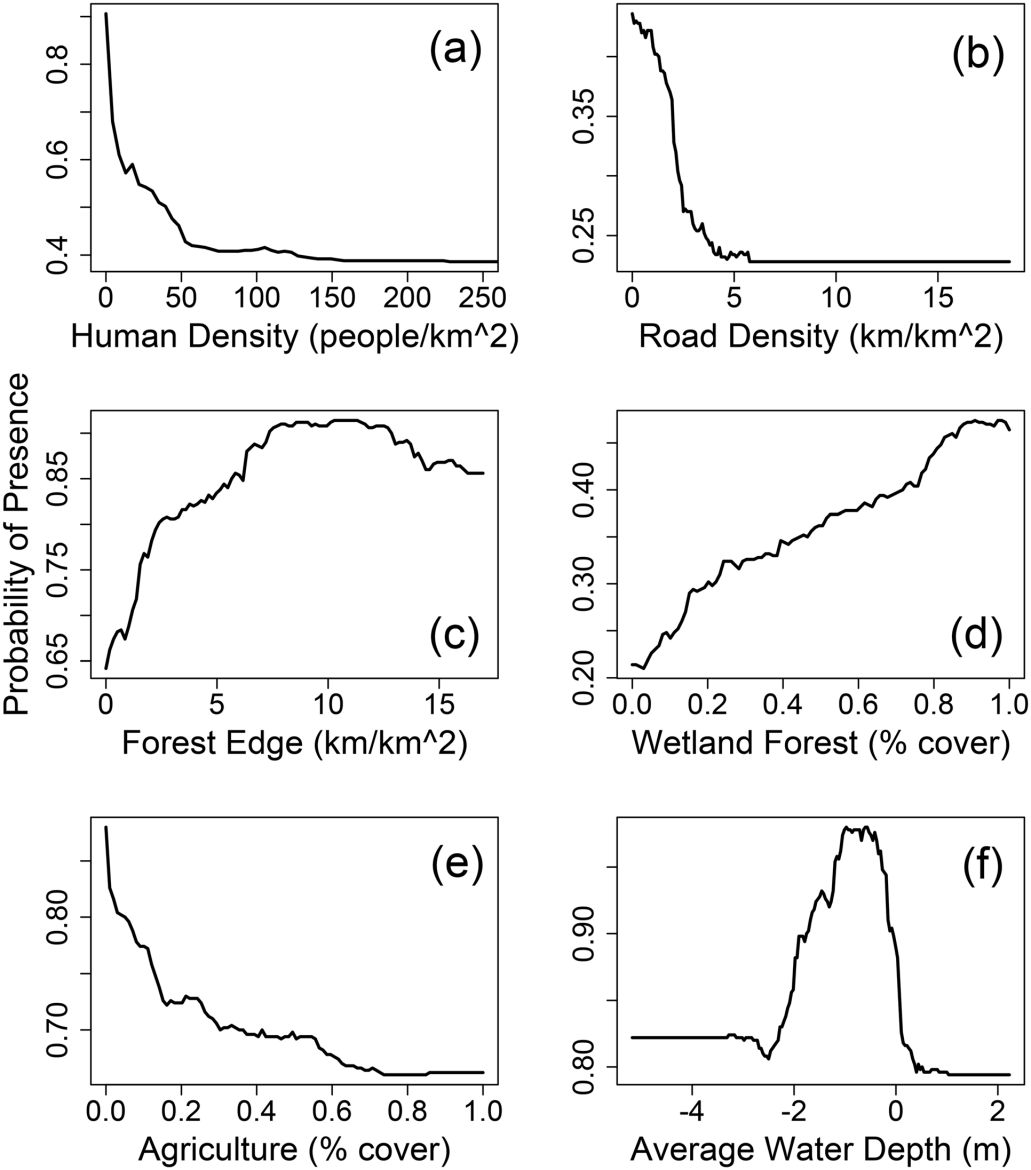
Sensitivity of model predictions (probability of presence, *P*) to changes in selected explanatory variables. (a) human population density; (b) road density; (c) forest edge; (d) wetland forest cover; (e) agriculture (other than pasture); (f) average dry season water depth. The response to each variable was examined at high, medium and low ranges of *P*. The *P* range where the variable had its largest effect is shown.

Sensitivity analysis results for six of the most important predictor variables are shown in Fig 5. Small increases in human density were predicted to have a pronounced negative effect on the probability of panther presence (*P*) (Fig 5a). In excellent (high *P* value) panther habitat, when human density increased from 0 to 10 people per km^2^, the model predicted a 0.3 decrease in the probability of panther use. At 50 people per km^2^, *P* decreased by almost 0.5. Likelihood of use by panthers continued to decrease up to about 140 people per grid cell, at which point further increases in human density had little effect. The human density variable had a similar but less pronounced effect on model outputs in lesser quality habitat. A related variable, road density was another strong negative predictor of panther presence. In medium quality habitat, a cell with no roads was predicted to be about twice as likely to support adult panthers than a cell with 5 km of roads (Fig 5b). Road density had its maximal effect at the middle ranges of *P*, but the effect was similar in all ranges. Since human population and roads generally occur together, the combined impact of increased roads and increased population density in residential developments, even low density developments, is predicted to be large.

The probability of panther presence was positively related to amount of forest edge, peaking at about 8 km of forest edge per cell (Fig 5c). Increasing forest edge from 0 to 8 km produced a corresponding increase of 0.36 in *P* in good quality panther habitat. The effect was similar but less pronounced in low *P* ranges. Beyond 8 km of forest edge, no further increase in *P* was observed. Increasing the amount of wetland forest cover in low quality habitat caused a steady increase in *P* from 0.21 up to 0.47 (Fig 5d). The increase was fairly constant throughout the entire range of forest coverage from 0 to 100%. The effect was similar at higher *P* ranges but began to drop off at about 80%.

Agricultural uses other than pasture within a grid cell were predicted to reduce its suitability for panthers, particularly in otherwise good (high *P*) habitat (Fig 5e). Panther presence was most likely when the average water level was just below ground surface. In high quality habitat, *P* was highest when the average water depth in the dry season was between -2 m and 0 m, and dropped off sharply on either side of this range. Peak probability of presence occurred at -0.6 m average water depth (Fig 5f). Average wet season water depths showed a similar probability profile. The other variables in the model had less profound or inconsistent effects. For example, increasing amounts of shrub (both upland and wetland) were predicted to have a positive effect on poor habitat and a negative effect on good habitat. Upland forest had a consistent positive effect but the maximum gain in *P* was less than 0.1.

**Fig 6:**
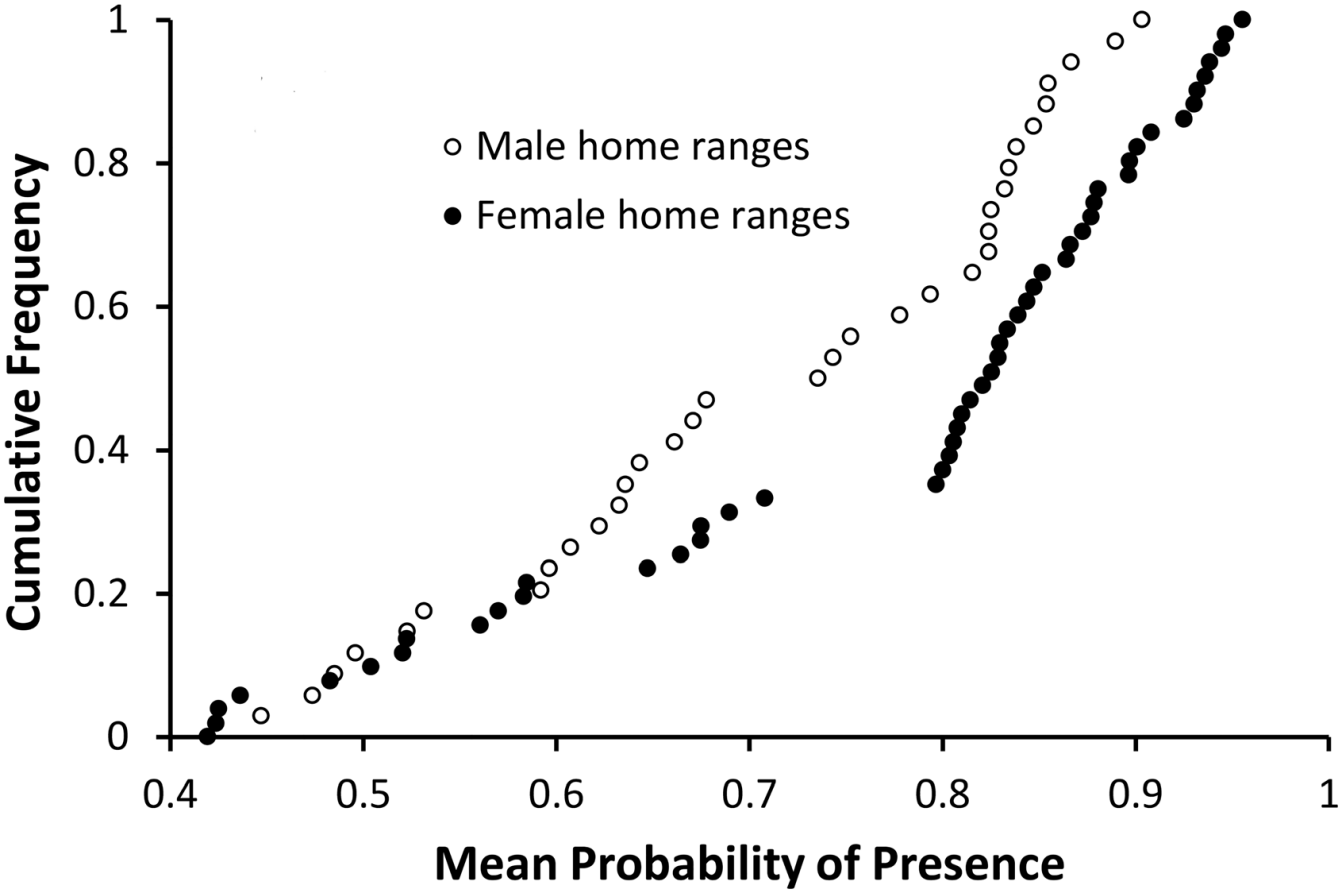
Average probability of presence in Florida panther home ranges. Males (open circles): median = 0.74, n = 35; females (solid circles): median = 0.82, n = 52. One male home range (average *P* = 0.35) is not shown.

The average probability of presence for each of the 87 panther home ranges was between 0.35 and 0.96, with a median value of 0.81. Except for one male with an unusually large home range, no panther home range had an average *P* below 0.4. Female panthers selected slightly higher quality habitat than males (p<0.01, Wilcoxon rank sum test) (Fig 6).

## Discussion

### Panther habitat

The most important factors determining panther presence or absence in a given cell were (1) the amount of forest cover, (2) human population density, (3) the amount of forest edge, and (4) the average water level (Fig 4). To our knowledge, this is the first model to demonstrate the importance of forest edge and water depth in panther habitat use, although other studies have examined related factors such as forest patch size [5, 7, 9, 36] and water regime (duration of flooding) [10]. A more widely applicable but still accurate model could probably be built based on the above four factors alone. However, with RF modeling when the objective is accurate prediction within the region, as was the case here, there is no compelling reason to reduce the number of variables. Our analysis showed that all variables made some contribution to model accuracy, however slight in some cases.

In the western states, the distribution of cougars is influenced by the amount of topographic heterogeneity and the quantity of forested cover [37], and in south Florida forested habitats have been shown to be important to panthers [4–9, 38], although it is topographically flat [39]. Maehr and Cox [5] and Maehr and Deason [36] asserted that Florida panthers used only forest patches >500 ha in size. However, Kautz et al. [7] and Onorato et al. [9] showed that forest patches of all sizes are used. Results from our study are consistent with the latter in that, related to forest patch size, the amount of forest edge was a highly predictive variable of adult panther presence. Studies by Holmes and Laundré [21] and Laundré and Loxterman [22] suggested that forest edges provide the necessary structural components for successful hunting by pumas, and Laundré and Hernández [40] concluded that use of edge areas allowed a puma to observe deer out in the open and to ambush deer as they moved between open and forest patches. Other studies have suggested that edge might be an important factor in panther habitat selection [5, 9]. Onorato et al. [9] reported often encountering panther kills in forests adjacent to more open habitats. We hypothesize that the importance of forest edge to panthers in south Florida is primarily as hunting and feeding habitat.

The extent of upland forest in our study area (895 km^2^) was small, and 333 km^2^ (37%) of that occurred outside the Primary Zone, in isolated patches surrounded by residential or agricultural land cover types. In addition, many areas used intensively by panthers within the Primary Zone contained very little land cover classified by FLUCCS as upland forest. Therefore, our model did not find upland forest cover to be a major factor in predicting presence of adult panthers on a landscape scale. However, upland forests ranked high in habitats selected *within* panther home ranges [6–9]. Benson et al. [41] found that panthers tended to select upland hardwoods, pinelands, and mixed wet forests for den sites. They suggested that the use of upland forests for denning may be a behavioral mechanism to maximize offspring survival in the flood-prone landscape of south Florida. However, panthers also selected mixed wet forests as den sites, did not avoid cypress swamps, and even denned in freshwater marsh [41]. Previous studies also found that panthers selected cypress swamps [6, 7]. Therefore, the importance of upland forest in panther habitat selection remains unclear, but it is obvious that forest cover in general is an essential element of panther habitat.

A consistent characteristic of panther den sites was extremely dense understory of saw palmetto (*Serenoa repens*), thickets, shrubs, or vines [17, 41]. Benson et al. [41] suggested that hydrology and resulting understory conditions in upland habitat types at least partially explain why pinelands and upland hardwoods were strongly selected by females as den sites. Our model showed that hydrology is indeed one of the most important factors determining the presence of adult panthers. The model indicated that the probability of adult panther presence is greatest when average water levels are just below the surface and drops off rapidly as water depths increase or decrease. Conditions would probably be optimal for the growth of dense understory vegetation when water depths are just below the surface. As water depths increasingly fall below the surface, however, understory vegetation may become less dense and, therefore, less usable to panthers. In addition, the areas within our study area where water depths were well below the surface were often associated with agricultural and urban land cover types.

Human land uses avoided by panthers on the landscape scale are also important in predicting panther presence or absence. Our model indicated that urbanized areas (as represented by human population and/or road density) were strong negative predictors of adult panther presence. The probability of adult panther presence dropped off precipitously as the number of people and roads per unit area increased (Fig 5a and 5b). The conversion of land for urban or agricultural uses eliminates, fragments, and alters panther habitat. Research by Burdett et al. [42] indicated that pumas avoided intensively developed suburban or urban areas, showed a negative response to exurban development (but individual responses were variable), and responded neutrally to rural development (again, individual responses were variable). In our model, agriculture (excluding pasture and rangelands) was of medium importance as a variable and had a pronounced negative effect on panther habitat.

### Comparison with previous landscape model

The boundary of the panther Primary Zone as drawn by Kautz et al. [7] was supported by our model, with a few notable exceptions. The Water Conservation Areas on the east side of the Primary Zone, the Shark River Slough in Everglades National Park, and the long, narrow corridor extending east from the Primary Zone and bisecting the Secondary Zone, do not contain adult panther habitat according to the probabilities assigned to those areas by our model (Fig 3b). These areas probably are used by transient males and fit more closely to the definition of the Secondary Zone [7]. The Shark River Slough portion, although not breeding panther habitat, is nevertheless an important connection between the main subpopulation to the north and the smaller Everglades subpopulation to the south, and thus represents an area that may be essential to panther survival and recovery. This area is currently protected within Everglades National Park, although rising water levels in this region could sever connections between the two subpopulations.

The RF model indicates that 5579 km^2^ of suitable adult panther habitat remain in southern Florida. Of this, 1399 km^2^ (25%) is in non-protected private ownership. Of the available breeding habitat, approximately 5232 km^2^ (93.8%) is contained within the Primary Zone defined by Kautz et al. [7], and 211 km^2^ (3.8%) is contained within their Secondary Zone. The remaining lands classified as adult habitat by our model (135.8 km^2^, 2.4%) are disjunct patches outside the Primary and Secondary zones and are seldom used by panthers, except for transient males (Fig 3b).

The Secondary Zone of Kautz et al. [7] is of little value to breeding panthers in its current state (Fig 3). Our model predicted an overall average probability of use of 0.086 for the Secondary Zone, compared with 0.455 for the Primary Zone. The former is much less than the minimum average value of a panther home range (0.352), suggesting that an adult panther could not establish a home range there. Kautz et al. [7] estimated that the effective area of the Secondary Zone is about 34.5% of that in the Primary Zone. In contrast, our model identified only 211 km^2^ of potential adult habitat in the Secondary Zone, compared with 5232 km^2^ in the Primary Zone (4.0%). Although containing little suitable habitat for adult, breeding panthers, the Secondary Zone is still important as a refuge for transient, non-breeding panthers. It also provides crucial connectivity to unoccupied areas and has the potential to be restored to more productive habitat.

Our study suggests that changes are needed to current conservation policies and practices for the Florida panther, especially with regard to methodologies for calculating habitat needs and impacts from development. For example, the U.S. Fish and Wildlife Service (USFWS) Panther Habitat Assessment Methodology (see Biological Opinions issued by USFWS since 2003 [43]) under-values the remaining adult habitat by overestimating the value of lands outside the Primary Zone. The USFWS methodology currently assumes lands in the Secondary Zone have a 69% equivalency with those in the Primary Zone. Our model shows that these lands, and a large portion of the Primary Zone itself, are of little value to support a breeding population of Florida panthers. As a result, compensation in the form of habitat protection required by the agency to offset losses due to development has been largely inadequate, because our study suggests that the amount of habitat remaining has been significantly overestimated. Even if all of the adult habitat within southern Florida had the maximum adult density of 2.80 panthers per 100 km^2^ as reported in Quigley and Hornocker [44], the total population would remain below 240 adults and subadults, a population size thought to be necessary to maintain genetic viability and a high probability of persistence [7]. Coupled with our findings, this indicates that there is not enough adult panther (breeding) habitat remaining in south Florida to maintain one genetically viable population.

## Conclusions

Our study has attempted to identify the remaining adult (breeding) habitat for the Florida panther south of the Caloosahatchee River. This population may already be at or close to carrying capacity, yet the panther population is probably below what is required for long-term genetic viability. Therefore, protection of the remaining breeding habitat in south Florida is essential to the survival and recovery of the subspecies and should receive the highest priority by regulatory agencies. Further loss of adult panther habitat is likely to reduce the prospects for survival of the existing population, and decrease the probability of natural expansion of the population into south-central Florida. This model is suitable for use by conservation agencies attempting to identify and protect the most valuable panther habitat in south Florida. Because it assigns a numerical “score” (probability of presence) to each square km in the study area, it will help managers to rank and prioritize those areas most important to panther survival. It will also be useful for calculating compensation for the inevitable habitat losses that will occur. One of the strong points of the model is its regional specificity for the unique south Florida landscape. However, it should be used with caution outside south Florida, due to the dominance of wetland habitats there compared to other areas.

## Supporting Information

S1 Table Landscape Data for South Florida Study Area. (CSV)

S1 Text Detailed Water Depth Methodology. (DOCX)
