# Supplementary material for: Landscape Analysis of Adult Florida Panther Habitat
Source: PLoS One. 2015 Jul 29;10(7):e0133044. doi: 10.1371/journal.pone.0133044 (PMC4519242; doi:10.1371/journal.pone.0133044)
Supplement: S1 Text — (DOCX) [file pone.0133044.s002.docx]

Introduction

This paper documents the method used to generate the spatial hydropatterns, *i.e.* average seasonal water depths used as hydrologic input for the panther regression model under development by the Fish and Wildlife Service. The purpose of the panther model is to allow resource managers to better understand how different climate change and sea level rise scenarios could change landscape scale hydropatterns which may have an effect on panther use of its’ primary habitat. Averaged wet and dry season water depths for the time period 1999 to 2009 were selected by species experts and modelers as a relevant statistic as input for each 1 mile by 1 mile grid cell of the model domain shown in Figure 1. A number of simplifying assumptions were required to overcome challenges to successfully generate hydropatterns over such a large landscape *i.e.,* greater than 3,500 square miles. The uncertainties associated with these assumptions, in addition to those associated with the topographic and hydrologic data themselves, the geostatistical methods used, commingling of ground water and surface water data, and the number of data points needed to represent the 10 year average is subsequently discussed.

Method

Creating a water depth surface is a relatively straightforward process that involves nothing more than subtracting ground surface elevation from a corresponding stage elevation (water level) both of which are referenced to the same horizontal and vertical datum. More specifically the steps to create a water depth surface include: 1) acquiring both hydrologic and topographic data referenced to the same vertical and horizontal datum; 2) reducing or summarizing the hydrologic data to the statistic of interest; 3) using geographic information or geostatistical software to generate surfaces derived from the hydrologic and topographic data; 4) converting these surfaces to a lattice of discretized point values at the required resolution and 5) subtracting each of the points of the ground surface elevation grid, from the associated stage grid *i.e.* water levels to produce water depth lattice. A flow diagram of the steps are shown in Figure 2 with a more detailed discussion on each element following in subsequent sections.

*Hydrology*

Daily mean surface and groundwater data, in feet National Geodetic Vertical Datum 1929 (NGVD) and horizontal data in North American Datum 1927 (NAD27) for the time period 1999 to 2009 and area shown in Figure 1 were acquired from the databases of Everglades National Park and the South Florida Water Management District (SFWMD), respectively DataForEver (DFE) and DBHYDRO. Stations both within and exterior to the model domain were included to minimize boundary or edge effects when generating the seasonal water depths. The 1999 to 2009 average wet and dry season average were calculated for each station. A 1999 to 2009 average for each station was only calculated if at least half of the years contained a seasonal value. Wet and dry season values were qualitatively evaluated in GIS for erroneous values. The error free average wet and dry season stage values were then used to generate separate water level raster images with a 1312 ft (400 m) cell resolution in ARC/GIS using the topo to raster utility and qualitatively evaluated for spurious results. This process was repeated until all obvious errors were removed. The final average wet and dry season raster images were converted to point feature shapefiles.

*Topography*

Topography or ground surface elevations used to calculate water depths originated from two sources, the United States Geological Survey (USGS) and a collaborative effort by the Corps of Engineers (COE) and SFWMD, shown in Figure 1 as red and blue hatched areas, respectively.

The USGS topography was collected and synthesized as part of the High Accuracy Elevation Data project (HAED). HAED collected elevation data in meters on a 1312 ft (400 m) topographic grid with a vertical accuracy of +/- 0.49 ft (15 centimeters) to define the topography in south Florida. The data were originally referenced to the horizontal datum North American Datum 1983 (NAD83) and vertical datum North American Vertical Datum 1988 (NAVD). Data for this project were collected using state-of-the-art GPS technology. In remote wetlands GPS technology with a helicopter-based instrument known as the Airborne Height Finder (AHF) was used to acquire elevations. In rural and urban areas a GPS station mounted on a vehicle linked to a high-quality dual frequency GPS receiver base station was used to establish a vertical and horizontal reference from which traditional survey methods could be used to acquire nearby ground surface elevations. These data were available to the Service in ARC/GIS format through the web (<http://sofia.usgs.gov/exchange/desmond/desmondelev.html>) and were post-processed to convert the ground surface elevation reported in meters to feet.

Topography for the western portion of the model domain was made available to the Service by the COE. The original data was provided as a raster image 100-ft resolution with elevation in feet. The data were referenced to NAD83 and NAVD88. Data used to generate the raster image were synthesized by the SFWMD and COE from numerous data sources, *i.e.* spot and contour elevations from elevation maps, photogrammetry, and points sampled by high-accuracy Light Detection and Ranging (LIDAR) technology, *etc.* as part of the Southwest Florida Feasibility Study. The range of vertical accuracy ranges from +/- 2.5 to +/- 0.3 ft, depending on the source. The raster image was re-sampled in ARC/GIS to a 1312 ft grid cell resolution using a bilinear interpolation method and converted to a point feature shapefile.

The two topographic point feature ARC/GIS shapefiles were merged by intersecting them with the panther model grid polygon feature shapefile. The resultant attribute information was exported to a *xyz* comma separated value (csv) file which was converted to NAD27 and NGVD29 using Corpscon 6.0 software developed by U.S. Army Topographic Engineering Center. These data were then imported back into ARC/GIS to create a point feature shapefile.

*Generating Average Seasonal Water Depth Hydropatterns*

The final steps in generating the average seasonal water depth required relating each stage value to a corresponding ground surface elevation, subtracting the ground surface elevation from the corresponding stages, and averaging the multiple water depths in each grid cell to produce a single value for each cell of the model. Because the origin, *i.e.* lower left of the bounding box for all the data sets were different and hence the points were not exactly aligned on top of one another a simplified approach to relate the stage to ground surface elevation points was required. This was achieved by applying 820 ft buffer to each average seasonal point feature shapefile and intersecting the result with the topographic point feature shapefile. The result was a point feature shapefile that contained a stage value and either 1, 2, or 3 corresponding ground surface elevations, in ft NGVD29 georeferenced by both panther model grid cell number and *xy* coordinates in NAD27. After additional QA/QC the resultant attribute information to verify that every cell contained a water depth value and there were no outliers the attributes were exported to a *xyz* comma separated value (csv) file for input directly into the panther regression model.


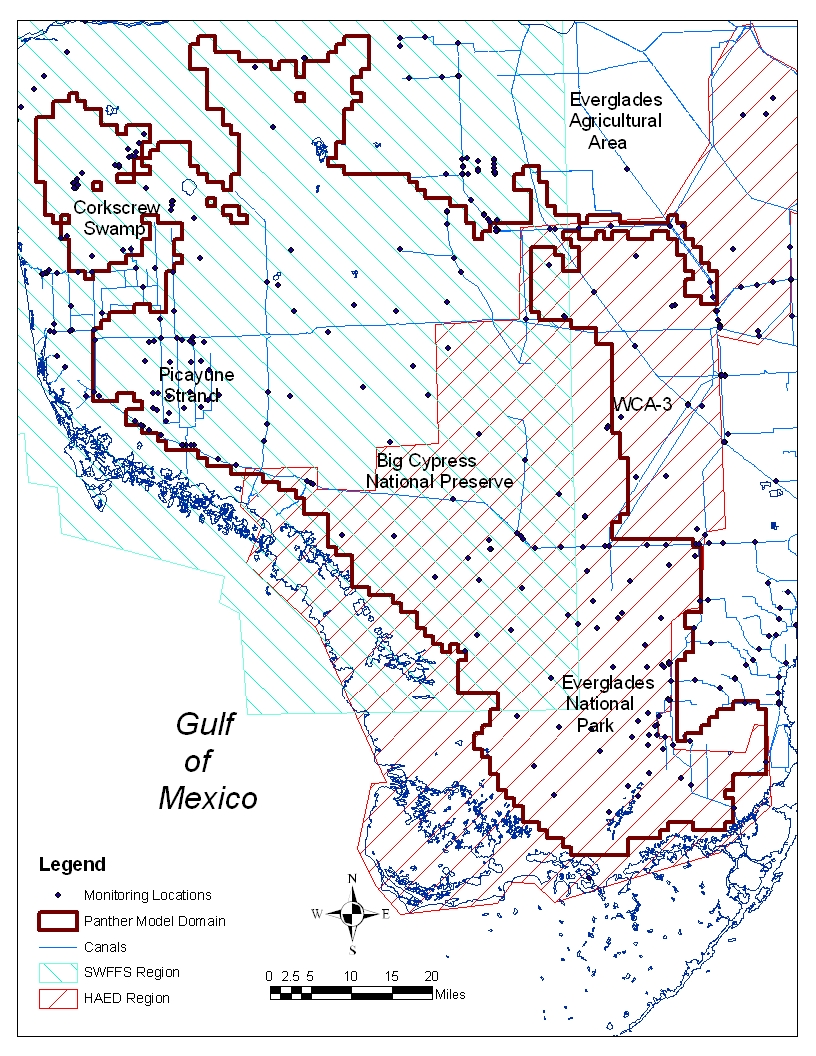


Figure 1 General location of panther model domain, monitoring stations used in the geo-statistical analysis, C&SF and SFWMD canals and levees, and regions of the two topographic datasets.

Figure 2 General steps used to create panther model hydrologic input data.
